# The IL-17A/Neutrophil axis plays a critical role in lethal infection induced by an emerging ultra-virulent *Streptococcus suis* serotype 5 strain

**DOI:** 10.1080/21505594.2026.2690810

**Published:** 2026-06-17

**Authors:** Guo Chen, Xiyan Zhang, Jianping Wang, Pengcheng Du, Hui Sun, Han Zheng, Jianguo Xu

**Affiliations:** a Microbiology and Clinical Laboratory, Mianyang Center for Disease Control and Prevention, Mianyang, China; b National Institute for Communicable Disease Control and Prevention, Chinese Center for Disease Control and Prevention, Beijing, China; c Medical Research Center, Beijing Institute of Respiratory Medicine and Beijing Chao-Yang Hospital, Capital Medical University, Beijing, China; d Key Laboratory of Coal Environmental Pathogenicity and Prevention, School of Public Health, Shanxi Medical University, Taiyuan, China; e Research Center for Reverse Microbial Etiology, Workstation of Academician, Shanxi Medical University, Taiyuan, China; f National Key Laboratory of Intelligent Tracking and Forecasting for Infectious Diseases, National Institute for Communicable Disease Control and Prevention, Chinese Center for Disease Control and Prevention,Beijing, China

**Keywords:** *Streptococcus suis*, serotype 5, ultra-virulent, moribund, IL-17A, neutrophils

## Abstract

*Streptococcus suis* (*S. suis*) is an important emerging zoonotic pathogen. In recent years, ultra-virulent serotype 5 clinical strains have emerged in China, characterized by causing high mortality in mice at early infection, even at low inoculation doses. In this study, we investigated the characteristics of lethal infection induced by the ultra-virulent *S. suis* serotype 5 strain SC2022MYS167 isolated from a patient with Streptococcal toxic shock-like syndrome (STSLS) and severe pneumonia. The lethal infection was associated with excessive bacterial loads and pro-inflammatory cytokines in peripheral blood and organs. Blocking IL-17A activity effectively reduced mouse mortality at infection stages 1 and 2. The lung tissues exhibited significantly higher levels of pro-inflammatory cytokines compared to liver and spleen tissues, with elevated IL-17A levels observed exclusively in the lung tissues of moribund mice at infection stage 1. CD11b^+^ Ly6G^+^ neutrophils recruited from peripheral blood significantly colocalized with IL-17A within lung tissues. Post-infection depletion of neutrophils rescued mice from lethal infection by significantly reducing the bacterial burden and levels of IL-17A, IL-6, and TNF-α in peripheral blood. A dose-dependent relationship was observed between neutrophil-depleting antibody and protection from lethal infection. These findings indicate the critical role of the IL-17A – neutrophil axis in STSLS development, pulmonary inflammation, pathological lesions, and subsequent acute host death induced by ultra-virulent serotype 5 strains, thereby providing a promising therapeutic target for reducing patient mortality.

## Introduction

*Streptococcus suis* (*S. suis*) is an important zoonotic pathogen that causes meningitis, septicemia, arthritis, pneumonia, endocarditis, shock, and even death in both pigs and humans [1–4]. Based on the distinguishing differences in capsular polysaccharide (CP) antigens, *S. suis* was classified into 29 known serotypes (1–19, 21, 23–25, 27–31, and 1/2). Although serotypes 2 and 14 predominate in human *S. suis* infections worldwide, zoonotic serotypes continue to emerge, including serotypes 1, 4, 5, 7, 9, 16, 24, and 31 [3,5–8]. Among these, serotype 5 represents the third most prevalent serotype in human *S. suis* infections [9]. To date, the research in pathogenicity has been mainly focused on serotype 2 strains which were characterized as epidemic (represented by the sequence type (ST) 7 strains), highly virulent (represented by the ST1 strains), and intermediately virulent (represented by the ST25 strains), with progressively decreasing virulence [10]. In the previous study, the substantial heterogeneity in virulence was revealed among *S. suis* serotype 5 population [9]. Notably, *S. suis* serotype 5 strain GX169 had been identified as the causative agent of streptococcal toxic shock-like syndrome (STSLS), with high mortality rates typically associated with *S. suis* serotype 2 epidemic strains [10,11]. Additionally, serotype 5 strains 2020WUSS080, 2020WUSS075, GX169, ID48908, and ID24665 demonstrated significantly higher virulence than serotype 2 highly virulent strain P1/7, characterized by increased early-phase mortality in infected mice at low infection doses [9]. Furthermore, the mechanism underlying lethal infection differed between the aforementioned serotype 5 strains and the serotype 2 highly virulent strain P1/7, due to significant differences in their virulence gene profiles [9]. In the study, *S. suis* serotype 5 strain SC2022MYS167 was isolated from a patient who died of STSLS complicated by severe pneumonia. The continued emergence of human infection cases caused by *S. suis* serotype 5 in China, coupled with limited information on its pathogenicity, highlights the urgent need for further investigation.

Understanding the characteristics of lethal infection induced by *S. suis* serotype 5 strains is crucial for developing effective treatments and preventing acute host responses. In this study, we first evaluated the virulence of *S. suis* serotype 5 strain SC2022MYS167 and subsequently investigated the bacterial loads, pro-inflammatory cytokine production, histopathological changes, and inflammatory receptor expression in peripheral blood and in organs of moribund mice infected with this strain. Particular focus was placed on dissecting the immunological mechanisms underlying the excessive cytokine response responsible for the acute host death at the early phase of infection.

## Materials and methods

### Human case description

On 27 July 2022, a 45-year-old male farmer was admitted to a hospital in Sichuan province, presenting with an acute onset of fever (42°C), chills, fatigue, palpitations, and muscle pain in both calves. The patient exhibited visible wounds on his hand. Upon admission, his vital signs were markedly abnormal: hypotension (blood pressure: 66/41 mmHg), tachycardia (heart rate: 116 bpm), and a respiratory rate of 20 breaths per minute. Laboratory tests indicated a leukocyte count of 10.77 × 10^9^/L, neutrophil ratio of 96.6%, platelet count of 26 × 10^9^/L, and procalcitonin level > 100 ng/mL. The patient’s coagulation profile revealed prolonged thrombin time (TT) of 37.5 seconds, prothrombin time (PT) of 29.5 seconds, and activated partial thromboplastin time (APTT) of 131.6 seconds. Liver function tests showed elevated levels of alanine transaminase (ALT) at 260.4 U/L, aspartate transaminase (AST) at 480.4 U/L, total bilirubin at 65.2 μmol/L, and direct bilirubin at 44.3 μmol/L. Markers of renal and cardiac dysfunction were also elevated, including blood urea nitrogen (UN: 18.26 mmol/L), creatinine (CR: 343 μmol/L), uric acid (714.5 μmol/L), lactate dehydrogenase (LDH: 972.8 U/L), and α-hydroxybutyrate dehydrogenase (HBDH: 421 U/L). Next-generation Capture sequencing (IDseq™Ultra, VISION MEDICALS, Guangzhou, China) detected 5,956 and 242,514 *S. suis* reads in the sputum and peripheral blood specimens, respectively. The *S. suis* strain was isolated from the patient’s peripheral blood sample and named SC2022MYS167. Based on clinical symptoms, signs, and laboratory results, the patient was diagnosed with STSLS induced by *S. suis* infection. One day after admission, the patient’s condition deteriorated rapidly, progressing to severe pneumonia and coma. The patient succumbed to multiple organ system failure on July 30.

### Bacterial strains, sequencing, bioinformatic, and antimicrobial susceptibility profiles analysis

In this study, the complete genome of *S. suis* strain SC2022MYS167 was sequenced according to previously described methods [8]. Species identification and serotype classification were confirmed through full-length *16S rRNA* gene sequences, pairwise average nucleotide identity (ANI) comparison with the *S. suis* type genome, and the presence of the serotype 5-specific *wzy* gene, respectively.

The multilocus sequence typing (MLST) and minimum core genome (MCG) typing of the SC2022MYS167 genome were performed using corresponding whole genome sequences in the PubMLST database (https://pubmlst.org/bigsdb?db=pubmlst_ssuis_seqdef) and the Pathogen Genome and Metagenome Analysis Cloud Platform (https://analysis.mypathogen.org/workflow/config/chinacdc/Ssuis_CGT/1/), respectively.

To investigate the phylogenetic position of SC2022MYS167, the *S. suis* serotype 5 population phylogeny constructed in our previous study was reconstructed by incorporating this strain, following the same phylogenetic pipeline and parameters as previously described [9].

The distribution of 154 known *S. suis* putative virulence-associated genes in strain SC2022MYS167 was investigated. Antibiotic resistance (AR) genes were identified by querying the ResFinder database (https://cge.food.dtu.dk/services/ResFinder/). Virulence-associated genes and AR genes with global match regions below 80% and amino acid sequence identity below 80% were considered absent. Intact mobile genetic elements (MGEs) carrying AR genes, including integrative and conjugative elements (ICEs) and prophages, were identified using previously described methods. The intact CP synthesis (*cps*) genes cluster was extracted from the genome, and the homology groups (HGs) of *cps* genes were assigned according to previously established nomenclature [12]. Sequence comparison of the *cps* gene cluster utilized the BLASTn algorithm with an e-value cutoff of e^−10^.

To determine whether the AR genes in genomes conferred the predicted resistance to the corresponding bacteria, we used the MIC-test strip (Liofilchem, Roseto degli Abruzzi, Italy) to assess the antimicrobial susceptibility of clindamycin (Cat. No. 920721, 0.016–256 μg/mL), erythromycin (Cat. No. 920511, 0.016–256 μg/mL), azithromycin (Cat. No. 920301, 0.016–256 μg/mL), tetracycline (Cat. No. 921141, 0.016–256 μg/mL), cefotaxime (Cat. No. 920431, 0.016–256 μg/mL), vancomycin (Cat. No. 920571, 0.016–256 μg/mL), penicillin (Cat. No. 921021, 0.016–256 μg/mL), and spectinomycin (Cat. No. 920141, 0.016–1024 μg/mL) according to the methods and resistance breakpoints previously described [9].

### Infection experiments

#### Survival assay

The virulence level of *S. suis* serotype 5 strain SC2022MYS167 was evaluated using a mouse infection model. For comparison, the *S. suis* serotype 2 epidemic ST7 strain SC84, known for enhanced pathogenicity [10], was included. Female C57BL/6 mice (6 weeks old) purchased from SiPeiFu Biotechnology (Beijing, PR China) received intraperitoneal injections of either a standard dose of 2 × 10^7^ CFU or a low dose of 2 × 10^6^ CFU of both strains in 1 mL of Todd-Hewitt broth (THB). To assess the contribution of specific cytokines and neutrophils to survival following SC2022MYS167 infection, mice received intraperitoneal injections of neutralizing antibodies one hour after low-dose challenge with SC2022MYS167. The following antibodies (Selleck, TX, USA) were administered: 200 μg anti – IFN-γ (Cat. No. A2105, Selleck, TX, USA) [13], 200 μg anti – IL-17A (Cat. No. A2120, Selleck) [14], or anti – Ly6G (Cat. No. A2158, Selleck) [15] at 300, 150, 100, or 50 μg, with each dose constituting a separate treatment group. The mock-infected group received 1 mL of THB only. Each infected group comprised 10 mice, while the mock-infected group contained five mice. All experiments were conducted independently twice, involving a total of 210 mice. Mortality rates were calculated using the Kaplan – Meier method. Ninety-six hours later, the survival mice were euthanized via cervical dislocation.

#### Bacterial loads, pro-inflammatory cytokines, and transcription analysis in moribund mice

Seven female C57BL/6 mice (6 weeks old) received intraperitoneal injections of a low dose of *S. suis* strain SC2022MYS167 in 1 mL of THB. The mock-infected group consisted of three mice receiving 1 mL of THB only. The experiment was conducted independently twice, involving a total of 20 mice. The moribund mice (characterized by lateral recumbency, convulsion, hypothermia, and shortness of breath) were euthanized via cervical dislocation during the period of 8 h post-infection, peripheral blood and organs (brain, lung, liver, spleen, and kidney) were aseptically collected.

The organs were precisely weighed, with half of each organ homogenized in 1 mL of PBS. Serial ten-fold dilutions of peripheral blood and homogenates were plated onto THB agar plates in duplicate for colony counting. Only plates containing well-separated and clearly distinguishable colonies (typically 30–300 colonies per spot) were counted. Sterile PBS was used as a negative control. All procedures were performed in a biosafety cabinet to prevent contamination. Results were expressed as CFU/mL for peripheral blood and CFU/0.1 g for organs.

The 200 μL homogenates of liver, spleen, and lung tissues underwent extraction and centrifugation for 5 min at 5,000×g at 2 to 8°C. The supernatants were collected and normalized to a protein concentration of 1 mg/mL using a bicinchoninic acid (BCA) protein assay kit (Cat. No. PC0020, Solarbio, Beijing, China). Concentrations of tumor necrosis factor-alpha (TNF-α) (Cat. No. 88–7324-88, Invitrogen, CA, USA), interleukin (IL)-6(Cat. No. 88–7064-88, Invitrogen), IL-17A (Cat. No. 88–7371-88, Invitrogen), IL-1β (Cat. No. 88–7013-88, Invitrogen), and interferon-gamma (IFN-γ) (Cat. No. 88–7314-88, Invitrogen) in both serum and tissue supernatants were measured using enzyme-linked immunosorbent assay (ELISA), following the manufacturer’s protocols. To ensure the reliability of cytokine measurements, each 96-well plate included duplicate standard curves, and all samples for a given cytokine were assayed on the same plate to avoid inter-plate variability. All samples were processed within 2 hours of collection, aliquoted, and stored at −80°C without any freeze – thaw cycles. Absorbance was read using a BioTek ELx808 microplate reader (Winooski, VT, USA) with a primary wavelength of 450 nm and a reference wavelength of 540 nm to correct for optical interference. Results were expressed as pg/mL for serum and pg/mg for tissues.

Total RNA was extracted from the remaining homogenates to analyze pro-inflammatory mediator and receptor gene transcription. Total RNA from liver, spleen, and lung tissues was isolated using Trizol reagent (Cat. No. 15596018CN, Invitrogen), and then 1 μg of total RNA was reverse transcribed in a 20 μL reaction system using kit from PrimeScript™ RT Master Mix (Cat. No. RR036A, Takara, Beijing, China). Each set of cDNA was diluted 10 times, and a 20 μL reaction system was composed of 2X TB Green Premix Ex Taq II FAST qPCR (Cat. No. CN830A, Takara), corresponding primers and ddH_2_0 followed by RT-PCR using Rotor-Gene Q instrument (QIAGEN, Thermo Fisher Scientific, USA). All reactions were performed at 95°C for 15 seconds, followed by 40 cycles of 95°C for 5 seconds and 60°C for 30 seconds. The mRNA expression levels were calculated using the 2^−ΔΔCt^ method with the *gapdh* gene as an internal reference. RNA from corresponding tissues of uninfected mice served as the calibration reference. The primer sequences used to detect the target genes are listed in Supplemental Table 1, which were synthesized by Beijing Tianyi Huiyuan Biotechnology Co., Ltd. Each 20 μL reaction contained 10 nM forward and reverse primers.

#### Analysis of histopathology of organs and the mechanism of IL-17A production within lung tissues of moribund mice

The other half of the organs was fixed for 24 h at room temperature in 4% buffered formalin. Following paraffin embedding, tissue sections were conventionally dewaxed, stained with hematoxylin and eosin (H&E), and dehydrated according to standard protocol. The images were acquired and analyzed using light microscopy. The expression of CD11b receptor in lung and liver sections was evaluated by immunohistochemistry (Servicebio, Hubei province, China). The lung and liver sections were blocked with 3% bovine serum albumin (BSA) dissolved in PBS for 30 min at room temperature and incubated with primary monoclonal anti-CD11b mouse antibody (Cat. No. GB15058, Servicebio, Wuhan, P. R. China) overnight at 4°C with dilution ratio of 1:200, followed by three rinses (5 min/each) in PBS. The sections were then incubated with an HRP-conjugated secondary antibody (Cat. No., GB23301, Servicebio) for 50 min at room temperature with dilution ratio of 1:200. After three additional PBS rinses (5 min/each), the sections were incubated with freshly prepared diaminobenzidine (DAB) staining solution. The coloring time was monitored under a light microscope. The cell nuclei were counterstained with hematoxylin for three minutes, followed by incubation with hematoxylin differentiation solution and blue-promoting solution. Sections were then subjected to a graded ethanol dehydration series (75%, 85%, and 100%) and cleared in n-butanol and xylene for 5 min each. The ratio of CD11b^+^ cells was calculated as the ratio of positively stained cells to the total number of cells in randomly selected microscopic fields.

To investigate the cellular source of IL-17A in lung tissue, immunofluorescence staining was performed to detect co-localization of CD11b, IL-17A, Ly6G, and F4/80 in lung tissue sections. Paraffin-embedded lung tissues underwent deparaffinization in xylene, dewaxing in ethanol, water bath repair in ethylenediaminetetraacetic acid (EDTA) (pH 9.0), and cooling to room temperature. The sections were blocked with 3% BSA for 30 min at room temperature and stained with rabbit CD11b antibody (Cat. No. GB15058, Servicebio) overnight at 4°C. Following incubation, Cy3-conjugated goat anti-rabbit immunoglobulin G (IgG) (Cat. No., GB21303, Servicebio) was applied as a secondary antibody and incubated for 50 min at room temperature with dilution ratio of 1:300. The sections were stained with tyramide signal amplification (TSA) dye for 10 min at room temperature while protected from light. The 1:3000 dilution ratio of IL-17A (Cat. No. GB11110-1, Servicebio), Ly6G (Cat. No. GB11229, Servicebio), or F4/80 (Cat. No. GB113373, Servicebio) antibodies were successively used to re-stain the sections following the aforementioned process. 4‘,6-diamidino-2-phenylindole (DAPI) was used for nucleus staining. The sections were then successively incubated with an Cy5-conjugated secondary antibody for Ly6G or F4/80 (Cat. No., GB27303, Servicebio), and FITC-conjugated secondary antibody for IL-17A (Cat. No., GB22403, Servicebio) for 50 min at room temperature with dilution ratio of 1:400 and 1:200, respectively. Sections were examined by fluorescence microscopy.

#### Analysis of the role of neutrophils in lethal infection induced by strain SC2022MYS167

To better define the contribution of neutrophils to lethal infection induced by strain SC2022MYS167, we included a neutrophil-depleted group treated with 100 μg anti – Ly6G antibody (Cat. No. A2158, Selleck) following low-dose infection with SC2022MYS167. Peripheral blood and lungs were aseptically collected from both the infection-only group and the anti – Ly6G-treated group at 5 h post-infection. Anti – Ly6G antibody was administered according to the protocol described in the survival assay. Each group contained five mice per experiment. The experiment was conducted independently twice, resulting in a total of 20 mice. For comparative analysis, bacterial burdens and IL-17A concentrations in peripheral blood and lung tissues from the two groups were quantified using the methods described above.

### Statistical analysis

Statistical analyses were performed using IBM SPSS Statistics version 22, and graphs were generated with GraphPad Prism 8. Female C57BL/6 mice (6 weeks old) were randomly assigned to different groups in each experiment. In the survival analysis, the personnel administering the injections were unaware of the contents of the syringes, which were prepared by another researcher. Histopathological evaluation, immunohistochemistry, and immunofluorescence analysis were all conducted under blinded conditions in which the evaluators were unaware of the group assignments. Blinding was not applied to other experiments due to the necessity of real-time procedural handling by investigators. No statistical methods were used to predetermine sample size. Estimates were made based on previous experience and sample sizes were kept at ten or less to adhere to 3 R (Replacement, Reduction, Refinement) standards, while remaining sufficient for statistical comparisons [16,17]. The sample size for each experiment is provided in the figure legends, and no data were excluded from the analyses. Data were first assessed for normality using the Shapiro-Wilk test. Comparisons between two groups with normally distributed data were conducted using Student‘s *t*-test (for analyses of transcriptional data, CD11b^+^ cell ratios, and Ly6G**^+^** cell ratios), whereas non-normally distributed data were analyzed using the Wilcoxon rank-sum test (for analyses of pro-inflammatory cytokine concentrations and bacterial burdens). Survival data were analyzed using the Gehan – Breslow – Wilcoxon test to compare differences among infection groups. A *p*-value < 0.05 was considered statistically significant for all analyses.

### Ethical approval

The epidemiological and clinical information of the patient infected with *S. suis* serotype 5 strain SC2022MYS167 was collected according to the requirements of the National Surveillance Program for Human Infection with *S. suis* (2009 Edition) issued by the Chinese Center for Disease Control and Prevention. In accordance with institutional policy, the study was exempted from ethical review by the Medical Ethics Committee of the Mianyang Center for Disease Control and Prevention.

All mice were bred and maintained in the Laboratory Animal Center of the Chinese Center for Disease Control and Prevention with free access to rodent chow and water. Mouse infection procedures followed the ARRIVE (Animal Research: Reporting of In Vivo Experiments) guidelines and were approved by the Laboratory Animal Welfare & Ethics Committee of the National Institute for Communicable Disease Control and Prevention, Chinese Center for Disease Control and Prevention (Approval code: 2023–047).

### Informed consent statement

The written informed consent, adhering to the Declaration of Helsinki, was obtained from the patient’s legal representative. Consent covered participation in the study and the publication of anonymized epidemiological and clinical information for research purposes. All necessary measures were taken to protect the patient’s identity.

## Results

### Genomic characteristics of serotype 5 strain SC2022MYS167

*S. suis* strain SC2022MYS167 was classified as ST1801 and clonal complex (CC) 1521. Although *S. suis* strain SC2022MYS167 was designated as MCG non-groupable (MCG group N) due to its clustering into MCG group 7–3 with a low proportion value of 23.08%, it demonstrated phylogenetic affinity with MCG group 3 genomes 40440 and 11538 in the phylogenetic tree of serotype 5 population (Supplemental Figure S1).

The distribution patterns of 154 known *S. suis* virulence genes were comparable between strain SC2022MYS167 and MCG group 3 genomes of *S. suis* serotype 5 population [9]. However, strain SC2022MYS167 contained the genes *epf* and *ofs* that were absent in all MCG group 3 genomes.

The serotype 5 strain SC2022MYS167 harbored *tet*(W), *erm*(B), and *ant(9)-Ia* genes, conferring resistance to tetracycline, Macrolide-Lincosamide-Streptogramin (MLS), and spectinomycin, respectively. These AR genes were located within the φSsuD.1-like prophage (87% coverage and 95% identity) integrated between the *rumA* (ACSEX2_06715) and *glf* (ACSEX2_06380) genes, designated as φSC2022MYS167.

The *cps* gene cluster of strain SC2022MYS167 exhibited pattern Ia and matched that of the serotype 5 reference genome 11538 (GenBank accession No. BR001003.1), except for a reversed arrangement of HG17, HG18, and HG19 in the 3’ region of the *cps* gene cluster.

### Antimicrobial susceptibility profile of *S. suis* serotype 5 strain SC2022MYS167

Strain SC2022MYS167 demonstrated susceptibility to cefotaxime, vancomycin, and penicillin. It exhibited resistance to spectinomycin, tetracycline, and showed concurrent resistance to clindamycin, erythromycin, and azithromycin (Supplemental Table 2). These findings confirmed that the AR genes conferred corresponding antibiotic resistance phenotypes to serotype 5 strain SC2022MYS167.

### Characteristics of lethal infection induced by *S.suis* serotype 5 strain SC2022MYS167

#### Ultra-virulent phenotype of strain SC2022MYS167

This study compared survival curves of C57BL/6 mice infected with serotype 5 strain SC2022MYS167 and serotype 2 epidemic strain SC84 at standard and low infection doses. Based on survival rate kinetics, the infection periods were classified as stage 1 (0–8 h post-infection), stage 2 (8–12 h post-infection), and stage 3 (12–16 h post-infection).

**Figure 1.**
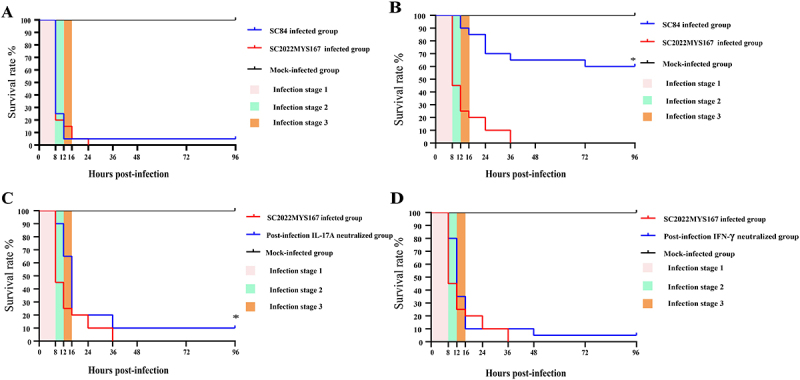
IL-17A contributes to the time-dependent and dose-independent lethal infection induced by *S. suis* serotype 5 ultra-virulent strain SC2022MYS167. Survival curves of C57BL/6 mice intraperitoneally infected with *S. suis* serotype 5 strain SC2022MYS167 and serotype 2 epidemic strain SC84 at standard dose (2 × 10^7^ CFU) (A) and low dose (2 × 10^6^ CFU) (B), respectively. Survival curves of mice infected with low-dose (2 × 10^6^ CFU) *S. suis* serotype 5 strain SC2022MYS167 and treated with neutralizing antibodies against IL-17A (C) or IFN-γ (D) (intraperitoneal injection at 1 h post-infection). Each infected group contained 10 mice, while the mock-infected group contained five mice. Each experiment was performed twice independently. Survival data were analyzed using the Kaplan – Meier method, and differences between groups were assessed using the gehan – Breslow – Wilcoxon test. *p* < 0.05 was considered significant. *: statistically significant difference (*p* < 0.05) in mortality compared to the low-dose of *S. suis* serotype 5 strain SC2022MYS167-infected group.

At the standard inoculum, both strains showed similar survival curves. At 96 h post-infection, survival rates were 0% for the SC2022MYS167 group and 5% for the SC84 group, with predominant mortality occurring during infection stage 1 (Figure 1(A)). In the low-dose SC84 group, survival rates increased significantly from 5% to 60% at 96 h post-infection, creating a marked difference between dose groups. Conversely, strain SC2022MYS167 showed no difference between dose groups, with complete mortality within 36 h post-infection in both cases. The primary difference was that low-dose SC2022MYS167 mortality shifted from stage 1 to stage 2, with 75% mortality during stage 2, while 90% of low-dose SC84-infected mice survived this period (Figure 1(B)). These observations suggest that the lethality of SC2022MYS167 is time-dependent but infection dose-independent. The serotype 5 strain SC2022MYS167 demonstrated higher pathogenicity than the epidemic SC84 strain. Based on the previous study [9], the criteria for ultra-virulent (UV) strains are further refined as: their virulence level is significantly higher than those of epidemic and highly virulent strains, with increased early‑phase mortality at low infection doses. Thus, *S. suis* serotype 5 strain SC2022MYS167 was classified as a UV strain in this study.

#### High bacterial loads in the peripheral blood and organs

Given that mortality in mice infected with a low dose of *S. suis* serotype 5 strain SC2022MYS167 primarily occurred during infection stage 1, peripheral blood and organ samples were collected from moribund mice during this stage. The first mortality was observed at 6 h post-infection, with eight moribund mice documented during infection stage 1.

**Figure 2.**
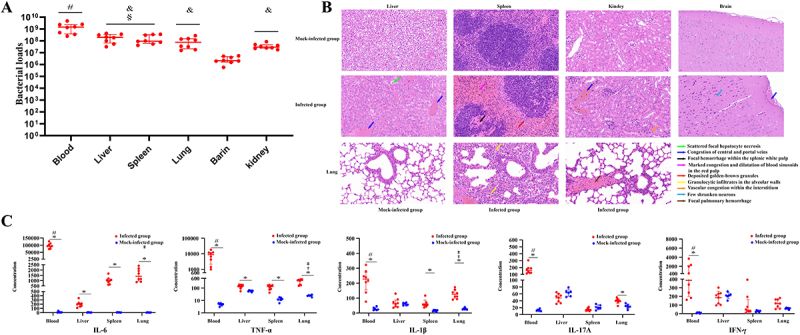
Characteristics of moribund mice infected with *S. suis* serotype 5 ultra-virulent strain SC2022MYS167 at infection stage 1. A. Bacterial loads in the peripheral blood and organs of moribund mice. Colony-forming units (CFUs) were quantified and expressed as CFU/mL for peripheral blood and CFU/0.1 g for organ tissues. Each infected group contained eight moribund mice. B. Representative histopathological images of organs from moribund mice. Hematoxylin and eosin (H&E) staining was performed; scale bar: 20 µm. Each infected group contained eight moribund mice, while the mock-infected group contained six mice. C. The levels of pro-inflammatory cytokines (IL-6, TNF-α, IL-1β, IL-17A, and IFN-γ) in serum and organs of moribund mice. Cytokine levels were expressed as pg/mL for serum and pg/mg for tissues. Each infected group contained eight moribund mice, while the mock-infected group contained six mice. Statistical differences in bacterial loads and pro-inflammatory cytokines between two groups were determined using the Wilcoxon rank-sum test. *p* < 0.05 was considered significant. #: significantly higher than those of tissues. §: significantly higher than those of kidney and brain tissues. &;: significantly higher than those of brain tissues. †: significantly higher than those of spleen tissues. ‡: significantly higher than those of liver tissues. *: significantly higher than those of mock-infected group.

The bacterial loads were quantified in the peripheral blood, liver, spleen, lung, kidney, and brain of moribund mice. The bacterial median count in peripheral blood reached 1.45 × 10^9^ CFU/mL, significantly exceeding that of the other organs. The viable median counts in liver, spleen, lung, kidney, and brain were 1.98 × 10^8^ CFU/0.1 g, 9.47 × 10^7^ CFU/0.1 g, 7.6 × 10^7^ CFU/0.1 g, 2.97 × 10^7^ CFU/0.1 g, and 2.14 × 10^6^ CFU/0.1 g, respectively. The bacterial loads in liver, spleen, and lung showed similar levels and were significantly higher than those in the brain which exhibited the lowest bacterial load among all organs. The bacterial load in kidney was significantly lower than that of the liver and the spleen (Figure 2(A)).

#### Organ-specific histopathological alterations

Notable histopathological changes were observed in the lungs, liver, and spleen of moribund mice. In the lungs, the most prominent lesions included mild to moderate thickening of alveolar septa, extensive neutrophil infiltration, perivascular edema, and localized hemorrhagic foci. The liver exhibited pronounced hepatocellular edema, marked vascular congestion within central and portal veins, and focal hepatocyte necrosis. Splenic tissues displayed mild hemorrhage within the white pulp, massive congestion and expansion of the red pulp, and lymphoid depletion. In contrast, the kidneys and brain exhibited only mild lesions. Renal changes were limited to slight renal interstitial vascular congestion and scattered eosinophils within the renal capsule. In the brain, occasional shrunken neurons were observed in the cortical regions (Figure 2(B)).

#### Excessive production of pro-inflammatory cytokines in the serum and organs

Strain SC2022MYS167 triggered excessive production of TNF-α, IL-6, IL-1β, IFN-γ, and IL-17A in the peripheral blood of moribund mice, with median values reaching 8697 pg/mL, 93,329 pg/mL, 228 pg/mL, 386 pg/mL, and 158 pg/mL, respectively. The levels of TNF-α, IL-6, IL-1β, IL-17A, and IFN-γ were also assessed in the lung, liver, and spleen tissue homogenates of moribund mice. Compared to mock-infected mice, TNF-α and IL-6 levels were significantly elevated in the liver, spleen, and lung tissues of moribund mice, while elevated IL-1β was observed only in the lung and spleen tissues. Notably, the lung tissues exhibited the highest levels of TNF-α and IL-1β, while similar IL-6 levels were found in the lung and spleen tissues, significantly exceeding those in the liver tissues. IFN-γ levels in the three organ tissue homogenates remained unchanged compared to mock-infected mice, while significant IL-17A production was observed exclusively in the lung tissues of moribund mice (Figure 2(C)).

### Mechanism of the lethal infection induced by the *S. suis* serotype 5 strain SC2022MYS167

#### IL-17A drives acute mortality in SC2022MYS167-infected mice

The roles of IFN-γ and IL-17A in acute death induced by serotype 5 strain SC2022MYS167 were evaluated through neutralization experiments. A marked difference in survival curves was observed between the strain SC2022MYS167 infected group and the corresponding IL-17A neutralization group, attributed to significantly decreased mortality in the IL-17A neutralization group during infection stages 1 and 2. IL-17A neutralization delayed the onset of death in serotype 5 strain SC2022MYS167 infected mice from infection stage 1 to stage 3 (Figure 1(C)). Conversely, no difference in survival curves was observed between the strain SC2022MYS167 infected group and the corresponding IFN-γ neutralization group (Figure 1(D)).

#### Organ-specific expression of innate immune receptors and inflammasomes

The activation of inflammasomes via Toll-like receptor 2 (TLR2) played crucial roles in the STSLS induced by the *S. suis* serotype 2 epidemic strains [18]. This study compared the mRNA expression levels of Toll-like receptors (TLRs) and inflammasomes in the livers, spleens, and lungs of moribund mice. *S. suis* serotype 5 strain SC2022MYS167 significantly elevated the expression of TLR2 and NOD-like receptor family pyrin domain containing 3 (NLRP3) mRNA in all three organs. The expression levels of TLR2 and NLRP3 mRNA in liver tissues were markedly higher compared to spleen and lung tissues, while spleen and lung tissues showed similar expression levels. Additionally, significantly higher expression levels of TLR1 and TLR6 mRNA were observed in lung and liver tissues, respectively. Compared to the mock-infected group, the expression levels of NOD-like receptor family pyrin domain containing 1 (NLRP1), NOD-like receptor family CARD domain containing 4 (NLRC4), and absent in melanoma 2 (AIM2) remained unchanged in all three tissues. A slight elevation in nucleotide-binding oligomerization domain-containing protein 1 (NOD1) mRNA expression was observed in liver tissues, while lung and spleen tissues showed no changes compared to the mock-infected group (Figure 3(A)).

**Figure 3A.**
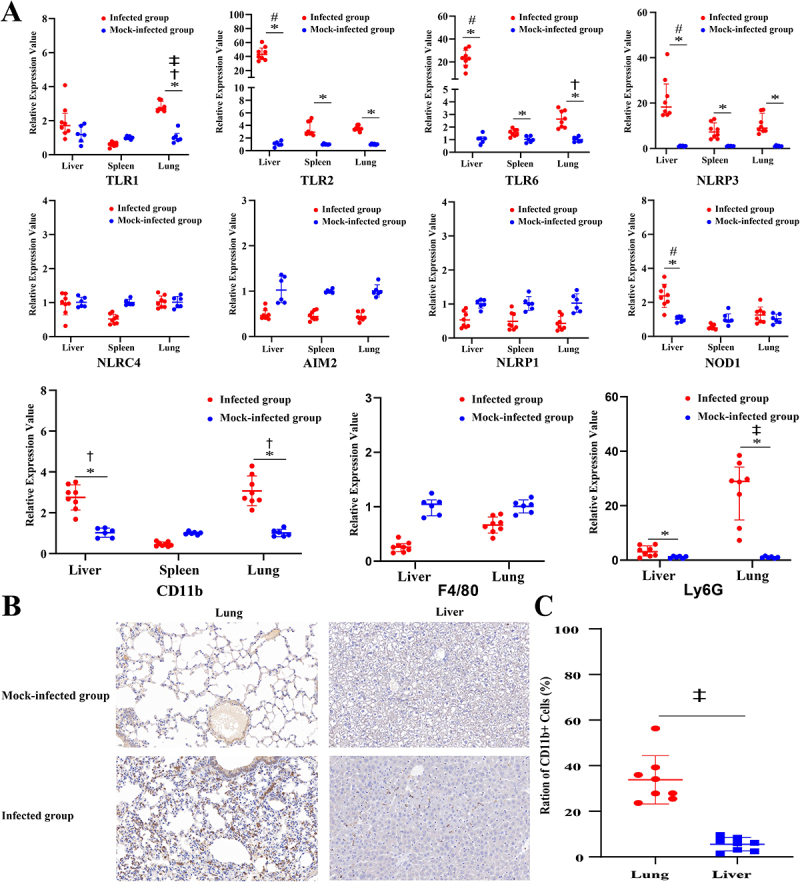
Expansion of CD11b^+^National Institute for Communicable Disease Control and Prevention, Chinese Center for Disease Control and Prevention, Beijing, ChinaNational Institute for Communicable Disease Control and Prevention, Chinese Center for Disease Control and Prevention, Beijing, Chinacells and their co-localization with IL-17A within lung tissues of moribund mice infected with *S. suis* serotype 5 ultra-virulent strain SC2022MYS167 at infection stage 1. A. The transcription levels of inflammatory receptors and cell surface markers in the liver, spleen, and lung tissues of moribund mice. Transcription levels were calculated after normalizing cycle thresholds against the “housekeeping” gene *gapdh* using the 2^−ΔΔCt^ method. Each infected group (moribund mice) contained eight mice, while the mock-infected group contained six mice. Statistical analyses were performed using an unpaired Student’s *t*-test. *: statistically significant difference between groups *(p* < 0.05); B. Immunohistochemical staining and quantification of CD11b^+^ cells in the lung and liver tissues. In immunohistochemical analysis, CD11b^+^ cells were stained brown by freshly prepared DAB, and cell nuclei were stained blue by hematoxylin. Scale bar: 20 µm. Each infected group contained eight moribund mice, while the mock-infected group contained six mice. C. The proportion ratio of CD11b^+^ cells in lung and live tissues. The values were calculated as the number of positively stained cells to the total number of cells. Each infected group contained eight moribund mice, while the mock-infected group contained six mice. Statistical analyses were performed using an unpaired Student’s *t*-test. D. Immunofluorescence analysis of CD11b^+^ cell co-localization with IL-17A, Ly6G, or F4/80 in the lung tissues of moribund mice. Fluorescent dyes were used to label CD11b (Cy3-red), IL-17A (FITC-green), Ly6G and F4/80 (Cy5-yellow), and nuclei (blue). Scale bar: 10 µm. Each infected group contained eight moribund mice, while the mock-infected group contained six mice. *p* < 0.05 was considered significant. *: significantly higher than those of mock-infected groups. #: significantly higher than those of spleen and lung tissues. †: significantly higher than those of spleen tissues. ‡: significantly higher than those of liver tissues.

#### Preferential accumulation of CD11b^+^ cells in lung tissues

qRT-PCR analysis revealed upregulation of CD11b mRNA expression within lung and liver tissues, while spleen tissues showed no significant change in CD11b mRNA expression levels (Figure 3(A)).

Despite similar mRNA levels of CD11b in lung and liver tissues, immunohistochemistry revealed significant differences in CD11b protein levels between these tissues (Figure 3(B)). The mean proportion ratio of CD11b^+^ cells within lung and liver tissues was 33.83% ± 10.6% and 5.56% ± 2.9%, respectively. The mean proportion ratio of CD11b^+^ cells in lung tissues was significantly higher than in liver tissues (Figure 3(C)).

**Figure 3B.**
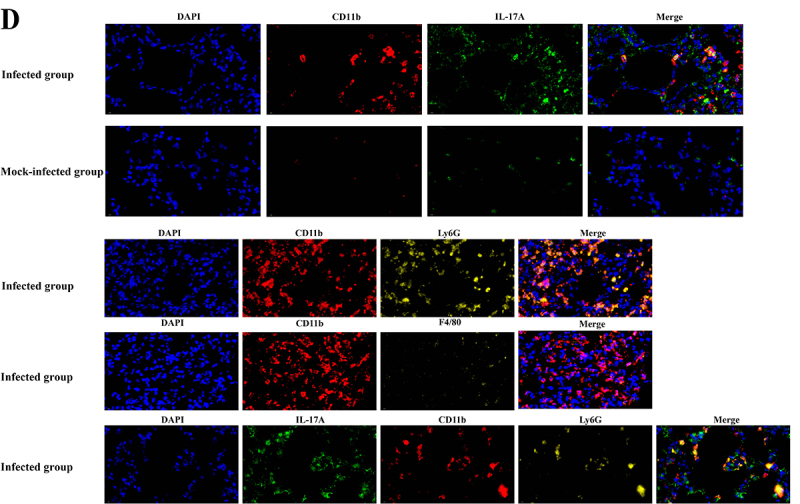
(Continued).

#### The CD11b^+^ cells were significantly co-localized with IL-17A within lung tissues

To investigate the correlation between IL-17A production and CD11b^+^ cells, lung tissues were labeled using CD11b and IL-17A antibodies. Immunofluorescence analysis demonstrated substantial co-localization of CD11b^+^ cells with IL-17A, indicating that CD11b^+^ cells were a primary cell type producing IL-17A within lung tissues (Figure 3(D)).

#### Neutrophils were the primary CD11b^+^ cell type in lung tissues

Further investigation of the cellular type of CD11b+ cells in lung tissues revealed that Ly6G mRNA expression was significantly higher in moribund mice than in the mock-infected group, while F4/80 mRNA expression remained unchanged. Moreover, Ly6G mRNA expression in the lung tissues of moribund mice was significantly higher than that in liver tissues (Figure 3(A)).

Immunofluorescence analysis demonstrated that most CD11b^+^ cells co-localized with Ly6G^+^ cells, while few CD11b^+^ cells co-localized with F4/80^+^ cells. To further confirm the correlation between Ly6G^+^ neutrophils and IL-17A, lung tissues were labeled with CD11b, IL-17A, and Ly6G antibodies. Results showed clear co-localization of IL-17A with CD11b^+^/Ly6G^+^ neutrophils (Figure 3(D)).

#### Lethal infection induced by UV strain SC2022MYS167 was improved by the appropriate depletion of neutrophils

**Figure 4.**
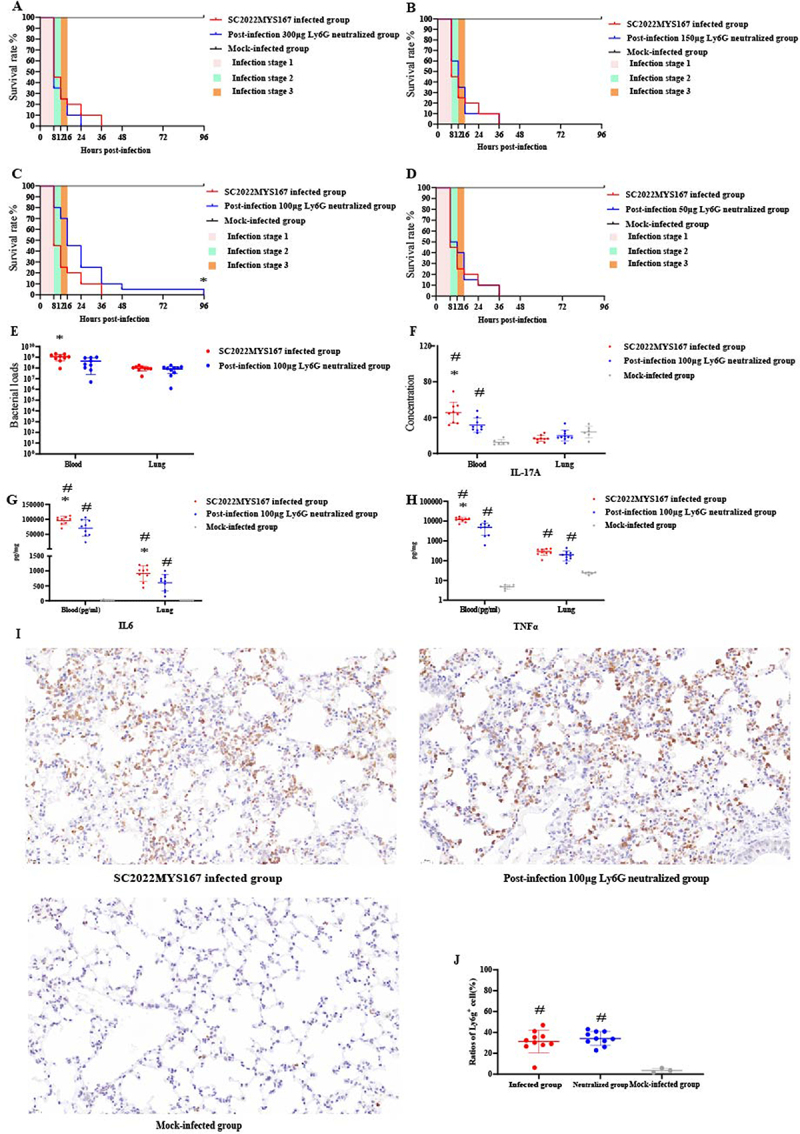
Neutrophils play a critical role in lethal infection induced by *S. suis* serotype 5 strain SC2022MYS167. A-D: survival of mice infected *S. suis* serotype 5 strain SC2022MYS167 and treated post infection with varying doses of anti – Ly6G antibodies (intraperitoneal injection at 1 h post-infection). Each infected group contained 10 mice, while the mock-infected group contained five mice. Each experiment was performed twice independently. Survival data were analyzed using the Kaplan – Meier method, and differences between groups were assessed using the gehan – Breslow – Wilcoxon test. *p* < 0.05 was considered significant. *: statistically significant difference between the group infected with a low dose of *S. suis* serotype 5 strain SC2022MYS167 and the group treated with 100 μg of anti-Ly6G antibody. E-H: bacterial loads (E) in peripheral blood, and the pro-inflammatory cytokines IL-17A (F), IL-6 (G), and TNF-α (H) concentrations in serum and lung tissues of different groups at 5 h post-infection. Cytokine levels were expressed as pg/mL in serum and pg/mg in lung tissues. Each infected group contained 10 mice, while the mock-infected group contained six mice. Statistical differences in bacterial loads and pro-inflammatory cytokines between two groups were determined using the Wilcoxon rank-sum test. *p* < 0.05 was considered significant. *: significantly higher than values in the low-dose SC2022MYS167-infected group. #: significantly higher than values in the mock-infected group. I. Immunohistochemical staining and quantification of Ly6G ^+^ cells in the lung tissues of different groups at 5 h post-infection. In immunohistochemical analysis, Ly6G^+^ cells were stained brown by freshly prepared DAB, and cell nuclei were stained blue by hematoxylin. Scale bar: 20 µm. Each infected group contained 10 mice, while the mock-infected group contained three mice. J. The proportion ratio of Ly6G^+^ cells in lung tissues of different groups at 5 h post-infection. The values were calculated as the number of positively stained cells to the total number of cells. Each infected group contained 10 mice, while the mock-infected group contained three mice. Statistical analyses were performed using an unpaired Student’s *t*-test. *p* < 0.05 was considered significant. #: significantly higher than values in the mock-infected group. Low-dose SC2022MYS167 group: *n* = 9 for all panels (one infected mouse was not included due to the death before 5 h). 100 μg anti-Ly6G group: *n* = 9 for peripheral blood data in E – H (the peripheral blood of one infected mouse was not collected due to the hypotension), *n* = 10 for all other data.

IL-17A, strongly correlated with neutrophils in this model, was identified as a critical mediator of lethality during *S. suis* strain SC2022MYS167 infection. To determine the roles of neutrophils in the lethal infection induced by UV strain SC2022MYS167, we depleted neutrophils by administering different doses of anti – Ly6G antibody after infection and compared survival among the dose groups. Mice treated with varying doses of anti-Ly6G antibody displayed distinct survival curves. At infection stage 1, mortality in the 300 μg anti – Ly6G antibody-treated group reached 65%, which was higher than 40%, 20%, and 50% observed in the 150 μg, 100 μg, and 50 μg groups, respectively. By the infection stage 3, mortality in the 300 μg, 150 μg, and 50 μg groups reached similar levels. Notably, only the 100 μg anti – Ly6G antibody-treated group showed a significant improvement in survival compared with the infected control group (Figure 4(A–D)).

#### Appropriate depletion of neutrophils reduced bacterial burden and inflammatory responses in peripheral blood

To assess how neutrophil depletion influences bacterial dissemination and inflammatory responses during lethal infection, we compared bacterial burdens and concentrations of IL-17A, IL-6, and TNF-α in peripheral blood and lung tissues between *S. suis* strain SC2022MYS167-infected mice and mice infected with SC2022MYS167 and treated post-infection with the protective 100 μg anti – Ly6G antibody.

At 5 h post-infection, significantly fewer bacteria were recovered from the peripheral blood of the 100 μg anti – Ly6G antibody-treated group than from the infected control group (Figure 4(E)). Serum IL-17A, IL-6, and TNF-α concentrations were also significantly reduced in the anti – Ly6G antibody-treated group (Figure 4(F,G,H)). By contrast, bacterial burdens (Figure 4(E)) and TNF-α level (Figure 4(H)) in lung tissues were similar between the two groups, and IL-17A level (Figure 4(F)) in lung tissues did not differ significantly from those in the mock-infected group for either condition. Notably, IL-6 concentration in lung tissues was significantly reduced in the anti – Ly6G antibody-treated group (Figure 4(G)).

Immunohistochemistry revealed significant accumulation of Ly6G^+^ cells in lung tissues of both groups compared with mock controls. The mean proportion of Ly6G^+^ cells in lung tissues was 31.0% ± 10.8% in the infected control group and 34.3% ± 6.6% in the post-infection 100 μg anti – Ly6G-treated group. The 100 μg anti – Ly6G antibody treatment did not reduce the neutrophil numbers in lung tissues (Figure 4(I,J)).

## Discussion

Serotype 5, an emerging zoonotic *S. suis* serotype, has emerged as the third most common serotype in human *S. suis* infection cases since 2007 [9], when the first human *S. suis* infection with serotype 5 caused by raw pork consumption was reported in Thailand [19]. In the phylogenetic tree of serotype 5 population, *S. suis* serotype 5 strain SC2022MYS167 did not cluster with other patient-derived strains, revealing significant genomic diversity among *S. suis* serotype 5 clinical strains. In addition, substantial heterogeneity in virulence was observed among *S. suis* serotype 5 strains. The repeated emergence of serotype 5 strains capable of causing high mortality in infected mice during the early phase of infection, even at low infection doses, highlights their potential to pose serious public health risks. The patient infected with *S. suis* serotype 5 ST1801 strain SC2022MYS167 developed STSLS, characterized by hypotension, thrombocytopenia, coagulation dysfunction, and multi-organ dysfunction. STSLS was predominantly reported in *S. suis* serotype 2 epidemic ST7 strains [10]. The 89K pathogenicity island (PAI) played a critical role in STSLS development induced by *S. suis* serotype 2 epidemic ST7 strains through the type IV secretion system (T4SS)-like system and two-component signal transduction systems *salR*/*salK* and *nisR*/*nisK* [20–23], which were absent in *S. suis* serotype 5 strain SC2022MYS167. Compared to the serotype 2 epidemic strain SC84 infected group, significantly higher early-phase mortality was observed in the serotype 5 strain SC2022MYS167 infected group at low infection doses. These findings indicated that the mechanism of serotype 5 strain SC2022MYS167 in inducing STSLS differed from that of serotype 2 epidemic strains. To effectively prevent and treat acute host death at the early phase of infection, clarification of the lethal infection features induced by *S. suis* serotype 5 UV strains is urgently needed.

The pro-inflammatory cytokine storm was crucial for STSLS development and subsequent acute host death [10,24]. Extremely high levels of pro-inflammatory cytokines were observed in the peripheral blood of moribund mice infected with *S. suis* serotype 5 strain SC2022MYS167, including TNF-α, IL-6, IL-1β, IFN-γ, and IL-17A. Previous research has demonstrated that exacerbated levels of IFN-γ, mainly secreted by over-activated natural killer (NK) cells, contributed to the acute death of mice infected with serotype 2 epidemic strain SC84 [24]. Although high levels of IFN-γ were observed in the peripheral blood of moribund mice, IFN-γ neutralization did not significantly reduce the mortality in serotype 5 strain SC2022MYS167 infected mice at the early phase of infection. Moreover, IFN-γ was not significantly induced in the liver, spleen, and lung tissues of moribund mice, indicating that IFN-γ might not play a critical role in the lethality induced by serotype 5 strain SC2022MYS167 during early infection. Conversely, blocking IL-17A activity effectively reduced mortality in infected mice, indicating the essential role of IL-17A in lethal infection induced by serotype 5 strain SC2022MYS167 during early infection.

The host of *S. suis* serotype 5 strain SC2022MYS167 developed severe pneumonia, with sputum samples revealing substantial *S. suis*-specific reads, indicating the crucial role of lung tissue inflammatory response in patient mortality. However, the roles of lungs in STSLS development induced by *S. suis* strains and subsequent acute host death remain insufficiently investigated. This study revealed that the lung tissues exhibited significantly higher levels of pro-inflammatory cytokines compared to liver and spleen tissues, with elevated IL-17A levels specifically observed in the lungs of moribund mice during infection stage 1. The inflammatory response pathways involved in lung tissues were different from those of liver tissues. Notably, these elevated cytokine levels in lungs were not attributable to differences in bacterial loads. Inflammatory factors frequently contribute to tissue damage during infectious pathogenesis. Varying degrees and types of histopathological changes were observed across lung, liver, and spleen tissues of moribund mice, with particular severity in the lungs. The findings suggest that lung pathologic lesions and pro-inflammatory cytokine secretion played important roles in the significant mortality rate of mice infected with *S. suis* serotype 5 strain SC2022MYS167 during the early infection.

This investigation examined the mechanisms underlying IL-17A production in lung tissues of moribund mice. *S. suis* serotype 2 epidemic strains activated NLRP3 inflammasome through Toll-like receptors to induce excessive IL-1β, subsequently inducing IL-17A expression in cell lines [18]. The enhanced Suilysin (SLY) expression of *S. suis* serotype 2 epidemic strain was crucial for NLRP3 inflammasome activation [18,25]. In contrast, infection with the serotype 5 strain SC2022MYS167 did not induce higher TLR2 or NLRP3 expression in lung tissues of moribund mice than those in liver or spleen tissues. Additionally, similar hemolytic activities were observed between serotype 2 epidemic strain SC84 and serotype 5 strain SC2022MYS167 *in vitro* (data were not shown). This suggests that the mechanism of IL-17A production in lung tissues of mice infected with serotype 5 strain SC2022MYS167 may differ from that of serotype 2 epidemic strain SC84.

Further investigation focused on the cellular source of IL-17A in lung tissues. IL-17A is a pro-inflammatory cytokine produced by various immune cells. In lungs, γδ T cells represent a primary source of early IL-17A production in several *in vivo* infection models [26]. Additionally, lung non-T cells were found to generate IL-17A [27]. Innate immunity provides immediate pathogen response through pro-inflammatory cytokine release. Given the significant IL-17A production during early infection, innate immune cells likely serve as important sources of rapid IL-17A production in response to serotype 5 strain SC2022MYS167 infection. CD11b, a granulocyte surface marker present on neutrophils, natural killer cells, and macrophages, represents an important innate immune cell population. Natural killer cells contribute to innate immune defense against pathogens primarily through IFN-γ release [24,28]. Neutrophils and macrophages constitute important IL-17A-producing populations [26,29]. Both human and mouse neutrophils demonstrate the capacity to secrete IL-17A [30]. In lung tissues, a notable increase in CD11b expression was observed at both mRNA and protein levels compared to liver and spleen tissues. IL-17A within lung tissue was found to co-localize with CD11b^+^ Ly6G^+^ neutrophils, which were substantially expanded in lung tissues of moribund mice. In contrast, the proportion of F4/80^+^ macrophages remained relatively stable and no co‑localization with CD11b cells was observed during infection stage 1. These findings suggest that neutrophils, rather than macrophages, were correlated with the production of IL-17A in the lung tissue of moribund mice at infection stage 1. The excessive recruitment of neutrophils contributes to the tissue damage during inflammation [31,32], and neutrophil depletion has been reported to significantly ameliorate clinical signs of central nervous system (CNS) disease following *S. pneumoniae* or *E. coli* infection [33,34]. To further investigate the role of neutrophils in the IL-17A production and lethal infection, neutrophils were depleted by administering different doses of anti – Ly6G antibody. In our SC2022MYS167 infection model, appropriate depletion of neutrophils was associated with significantly promoted survival, accompanied by reduced bacterial burdens and attenuated inflammatory cytokines IL-17A, IL-6, and TNF-α in peripheral blood at the early phase of infection. This observation demonstrated that neutrophils participated in the IL-17A production in this model. Notably, the excessive depletion of neutrophils led to harmful consequences in this study, further complicating the roles of neutrophils in the lethal infection.

Neutrophils are central effectors of innate immunity [35,36] and play important roles during the infection. Neutrophils are composed of heterogenous subpopulations with distinct functions in the infection, including immunosuppressive and proinflammatory functions [37,38]. Immunosuppressive neutrophils contribute to pathogen control during bacterial infections through phagocytosis, the extracellular toxic effect, production of reactive oxygen species (ROS), and release neutrophil extracellular traps (NETs) [32]. The proinflammatory neutrophils possess an enhanced ability to release proinflammatory cytokines [37,39], while the immunosuppressive neutrophils can efficiently suppress CD4^+^ and CD8^+^ T cell proliferation [37,40]. The proportions of activated subpopulations with distinct functions varied across bacterial infections. Thus, the contribution of neutrophils to host defense and pathology is highly context-dependent [33,34,36]. For instance, the capacity of *S. suis* strains to induce NET release from neutrophils depended on their serotypes and sequence types (STs). The *S. suis* ST28 (serotype 2) and ST1173 (non‑typeable) strains failed to induce NET release from neutrophils, whereas the ST1 (serotype 2) and ST7 (serotype 2) strains induced strong NET production [41,42]. The contribution of neutrophils to host survival by reducing bacterial burdens has also been demonstrated in infection with the *S. suis* ST1 strain [43]. In our unpublished data, the *S. suis* serotype 2 epidemic strain (ST7) induced higher NET production *in vivo* than did the serotype 7 UV strain (ST373), while the bacterial loads in mice infected with the serotype 7 strain were significantly higher than those in mice infected with the serotype 2 epidemic strain. Consistent with this interpretation, numerous studies have also demonstrated that neutrophils have a poor capacity to eliminate *S. suis* strains [23,42,44,45]. Until now, studies investigating neutrophil-driven immunopathology in *S. suis* infection remain limited. Appropriate depletion of neutrophils resulted in the attenuated inflammatory cytokines in peripheral blood at the early phase of infection. Therefore, we proposed that neutrophils mainly exerted pro-inflammatory functions mainly by releasing excessive inflammatory cytokines, thereby exacerbating the lethal infection induced by *S. suis* serotype 5 UV strain SC2022MYS167.

Previous research has documented the effects of TNF-α and IL-17A in neutrophil recruitment [46–48]. The synergistic chemotactic effect of TNF-α and IL-17A in lung tissues suggested that the substantial expansion of neutrophils in lung tissues originated from peripheral blood at the early infection phase. In addition, IL-17A receptors are broadly expressed on neutrophils, which are capable of responding to IL-17A [30,49].

Given the positive association between IL-17A production and neutrophil accumulation, we propose the existence of an IL-17A/neutrophil axis that played a critical role in the lethal infection induced by serotype 5 strain SC2022MYS167. The cross-regulation of neutrophils and IL-17A in the lethal infection is reflected in the following aspects: i. Neutrophils participate in the IL-17A production. ii. The robust production of IL-17A contributes to neutrophil recruitment to the peripheral blood and lung tissues of moribund mice at the early phase of infection. iii. IL-17A contributes to neutrophils releasing excessive cytokines such as IL-6 and TNF-α, thereby accelerating the progression of STSLS. Appropriate depletion of neutrophils reduced the proportion of pro-inflammatory neutrophils and alleviated the inflammatory response by disrupting the feedback loop between IL-17A and neutrophils. The ameliorated immune environment may enable immunosuppressive neutrophils to produce ROS and NETs, thereby reducing the bacterial loads in peripheral blood.

In contrast to findings in peripheral blood, anti – Ly6G antibody treatment reduced only IL-6 production and had no effect on TNF-α production, bacterial burden, and proportion of neutrophils in lung tissues during the early phase of infection. One possible explanation is that the markedly increased neutrophil population in peripheral blood preferentially consumed the circulating anti-Ly6G antibody, resulting in minimal depletion of lung-infiltrating neutrophils, whereas only the early production of IL-6 was affected. To determine the optimal timing for neutrophil depletion, future studies should systematically characterize the kinetics of neutrophil expansion in peripheral blood and lung tissues following infection.

Our findings suggest that infection with the *S. suis* serotype 5 strain SC2022MYS167 leads to marked activation of the IL-17A/neutrophil axis, which in turn drives excessive inflammation and contributes substantially to STSLS development, pulmonary inflammation and pathological lesions, ultimately leading to rapid death of the host.

In conclusion, this study elucidated the characteristics of lethal infection induced by the *S. suis* serotype 5 UV strain SC2022MYS167. A key finding was the uncontrolled IL-17A/neutrophil feedback loop, which may play critical roles in the development of STSLS and subsequent acute death of the infected mice. These findings provide valuable insights for reducing mortality effectively.

## Supplementary Material

Supplemental Material

Author Checklist.pdf

Supplemental Table 2.docx

supplementary file clean document.docx

Supplemental Table 1.docx

## Data Availability

The complete genome sequence of SC2022MYS167 was deposited in the GenBank database under the accession number CP195773. The data that support the findings of this study and the supplementary materials are openly available in Figshare at https://doi.org/10.6084/m9.figshare.30995566 [50].

## References

[1] Liu P, Zhang Y, Tang H, et al. Prevalence of *Streptococcus suis* in pigs in China during 2000–2021: a systematic review and meta-analysis. One Health. 2023;16:100513. doi: 10.1016/j.onehlt.2023.100513 37363255 PMC10288055

[2] Hlebowicz M, Jakubowski P, Smiatacz T. *Streptococcus suis* meningitis: epidemiology, clinical presentation and treatment. Vector Borne Zoonotic Dis. 2019;19(8):557–17. doi: 10.1089/vbz.2018.2399 30855223

[3] Dutkiewicz J, Sroka J, Zając V, et al. *Streptococcus suis*: a re-emerging pathogen associated with occupational exposure to pigs or pork products. Part I – epidemiology. Ann Agric Environ Med. 2017;24(4):683–695. doi: 10.26444/aaem/79813 29284248

[4] Goyette-Desjardins G, Auger JP, Xu J, et al. *Streptococcus suis*, an important pig pathogen and emerging zoonotic agent—an update on the worldwide distribution based on serotyping and sequence typing. Emerg Microbes Infect. 2014;3(1):1–20. doi: 10.1038/emi.2014.45 PMC4078792 26038745

[5] Segura M, Aragon V, Brockmeier SL, et al. Update on *Streptococcus suis* research and prevention in the era of antimicrobial restriction: 4th international workshop on *S. suis*. Pathogens. 2020;9(5):374. doi: 10.3390/pathogens9050374 32422856 PMC7281350

[6] Hatrongjit R, Fittipaldi N, Jenjaroenpun P, et al. Genomic comparison of two *Streptococcus suis* serotype 1 strains recovered from porcine and human disease cases. Sci Rep. 2023;13(1):5380. doi: 10.1038/s41598-023-32724-z 37009816 PMC10068604

[7] Wang X, Sun J, Bian C, et al. The population structure, antimicrobial resistance, and pathogenicity of *Streptococcus suis* cps31. Vet Microbiol. 2021;259:109149. doi: 10.1016/j.vetmic.2021.109149 34147764

[8] Liang P, Wang M, Gottschalk M, et al. Genomic and pathogenic investigations of *Streptococcus suis* serotype 7 population derived from a human patient and pigs. Emerg Microbes Infect. 2021;10(1):1960–1974. doi: 10.1080/22221751.2021.1988725 34635002 PMC8525962

[9] Zhang X, Zhu J, Kerdsin A, et al. *Streptococcus suis* serotype 5: emerging zoonotic threat with distinct genomic heterogeneity. Virulence. 2025;16(1):2523882. doi: 10.1080/21505594.2025.2523882 40574283 PMC12218517

[10] Ye C, Zheng H, Zhang J, et al. Clinical, experimental, and genomic differences between intermediately pathogenic, highly pathogenic, and epidemic *Streptococcus suis*. J Infect Dis. 2009;199(1):97–107. doi: 10.1086/594370 19016627

[11] Yu H, Jing H, Chen Z, et al. Human *Streptococcus suis* outbreak, Sichuan, China. Emerg Infect Dis. 2006;12(6):914–920. doi: 10.3201/eid1206.051194 16707046 PMC3373052

[12] Okura M, Takamatsu D, Maruyama F, et al. Genetic analysis of capsular polysaccharide synthesis gene clusters from all serotypes of *Streptococcus suis*: potential mechanisms for generation of capsular variation. Appl Environ Microbiol. 2013;79(8):2796–2806. doi: 10.1128/aem.03742-12 23416996 PMC3623174

[13] Li Q, Lv K, Jiang N, et al. Sod3 suppresses early cellular immune responses to parasite infection. Nat Commun. 2024;15(1):4913. doi: 10.1038/s41467-024-49348-0 38851821 PMC11162418

[14] Huang D, Wang Y, Zhai X, et al. Scleroglucan protects the intestine from irradiation-induced injury by targeting the IL-17 signaling pathway. Int Immunopharmacol. 2024;129:111614. doi: 10.1016/j.intimp.2024.111614 38350358

[15] Olofsen PA, Stip MC, Jansen JHM, et al. Effective, long-term, neutrophil depletion using a murinized anti-ly-6G 1A8 antibody. Cells. 2022;11(21):3406. doi: 10.3390/cells11213406 36359801 PMC9656769

[16] Roje B, Zhang B, Mastrorilli E, et al. Gut microbiota carcinogen metabolism causes distal tissue tumours. Nature. 2024;632(8027):1137–1144. doi: 10.1038/s41586-024-07754-w 39085612 PMC11358042

[17] Kang W, Wang M, Yi X, et al. Investigation of genomic and pathogenicity characteristics of *Streptococcus suis* ST1 human strains from Guangxi Zhuang autonomous region (GX) between 2005 and 2020 in China. Emerg Microbes Infect. 2024;13(1):2339946. doi: 10.1080/22221751.2024.2339946 38578304 PMC11034456

[18] Lin L, Xu L, Lv W, et al. An NLRP3 inflammasome-triggered cytokine storm contributes to streptococcal toxic shock-like syndrome (STSLS). PLOS Pathog. 2019;15(6):e1007795. doi: 10.1371/journal.ppat.1007795 31170267 PMC6553798

[19] Kerdsin A, Dejsirilert S, Sawanpanyalert P, et al. Sepsis and spontaneous bacterial peritonitis in Thailand. Lancet. 2011;378(9794):960. doi: 10.1016/s0140-6736(11)60923-9 21890062

[20] Xu J, Fu S, Liu M, et al. The two-component system NisK/NisR contributes to the virulence of *Streptococcus suis* serotype 2. Microbiol Res. 2014;169(7–8):541–546. doi: 10.1016/j.micres.2013.11.002 24342108

[21] Zhao Y, Liu G, Li S, et al. Role of a type IV-like secretion system of *Streptococcus suis* 2 in the development of streptococcal toxic shock syndrome. J Infect Dis. 2011;204(2):274–281. doi: 10.1093/infdis/jir261 21673039

[22] Li M, Shen X, Yan J, et al. Gi-type T4SS-mediated horizontal transfer of the 89K pathogenicity island in epidemic *Streptococcus suis* serotype 2. Mol Microbiol. 2011;79(6):1670–1683. doi: 10.1111/j.1365-2958.2011.07553.x 21244532 PMC3132442

[23] Li M, Wang C, Feng Y, et al. Salk/Salr, a two-component signal transduction system, is essential for full virulence of highly invasive *Streptococcus suis* serotype 2. PLOS ONE. 2008;3(5):e2080. doi: 10.1371/journal.pone.0002080 18461172 PMC2358977

[24] Lachance C, Gottschalk M, Gerber PP, et al. Exacerbated type II interferon response drives hypervirulence and toxic shock by an emergent epidemic strain of *Streptococcus suis*. Infect Immun. 2013;81(6):1928–1939. doi: 10.1128/iai.01317-12 23509145 PMC3676015

[25] Song L, Li X, Xiao Y, et al. Contribution of Nlrp3 inflammasome activation mediated by suilysin to streptococcal toxic shock-like syndrome. Front Microbiol. 2020;11:1788. doi: 10.3389/fmicb.2020.01788 32922370 PMC7456889

[26] Cua DJ, Tato CM. Innate IL-17-producing cells: the sentinels of the immune system. Nat Rev Immunol. 2010;10(7):479–489. doi: 10.1038/nri2800 20559326

[27] Mei J, Liu Y, Dai N, et al. Cxcr2 and Cxcl5 regulate the IL-17/G-CSF axis and neutrophil homeostasis in mice. J Clin Invest. 2012;122(3):974–986. doi: 10.1172/jci60588 22326959 PMC3287232

[28] Goldmann O, Chhatwal GS, Medina E. Contribution of natural killer cells to the pathogenesis of septic shock induced by *Streptococcus pyogenes* in mice. J Infect Dis. 2005;191(8):1280–1286. doi: 10.1086/428501 15776374

[29] Song C, Luo L, Lei Z, et al. Il-17-producing alveolar macrophages mediate allergic lung inflammation related to asthma. J Immunol. 2008;181(9):6117–6124. doi: 10.4049/jimmunol.181.9.6117 18941201

[30] Taylor PR, Roy S, Leal SM, et al. Activation of neutrophils by autocrine IL-17A-IL-17RC interactions during fungal infection is regulated by IL-6, IL-23, RORγt and dectin-2. Nat Immunol. 2014;15(2):143–151. doi: 10.1038/ni.2797 24362892 PMC3972892

[31] Camicia G, Pozner R, de Larrañaga G, et al. Neutrophil extracellular traps in sepsis. Shock. 2014;42(4):286–294. doi: 10.1097/shk.0000000000000221 25004062

[32] Bleuzé M, Gottschalk M, Segura M. Neutrophils in *Streptococcus suis* infection: from host defense to pathology. Microorganisms. 2021;9(11):2392. doi: 10.3390/microorganisms9112392 34835517 PMC8624082

[33] Ribes S, Regen T, Meister T, et al. Resistance of the brain to Escherichia coli K1 infection depends on MyD88 signaling and the contribution of neutrophils and monocytes. Infect Immun. 2013;81(5):1810–1819. doi: 10.1128/iai.01349-12 23478323 PMC3648016

[34] Mildner A, Djukic M, Garbe D, et al. Ly-6G+CCR2- myeloid cells rather than Ly-6ChighCCR2+ monocytes are required for the control of bacterial infection in the central nervous system. J Immunol. 2008;181(4):2713–2722. doi: 10.4049/jimmunol.181.4.2713 18684962

[35] Moffat A, Gwyer Findlay E. Evidence for antigen presentation by human neutrophils. Blood. 2024;143(24):2455–2463. doi: 10.1182/blood.2023023444 38498044

[36] Kolaczkowska E, Kubes P. Neutrophil recruitment and function in health and inflammation. Nat Rev Immunol. 2013;13(3):159–175. doi: 10.1038/nri3399 23435331

[37] Silvestre-Roig C, Fridlender ZG, Glogauer M, et al. Neutrophil diversity in health and disease. Trends Immunol. 2019;40(7):565–583. doi: 10.1016/j.it.2019.04.012 31160207 PMC7185435

[38] Deniset JF, Kubes P. Neutrophil heterogeneity: bona fide subsets or polarization states? J Leukoc Biol. 2018;103(5):829–838. doi: 10.1002/jlb.3ri0917-361r 29462505

[39] Tecchio C, Micheletti A, Cassatella MA. Neutrophil-derived cytokines: facts beyond expression. Front Immunol. 2014;5:508. doi: 10.3389/fimmu.2014.00508 25374568 PMC4204637

[40] Scapini P, Marini O, Tecchio C, et al. Human neutrophils in the saga of cellular heterogeneity: insights and open questions. Immunol Rev. 2016;273(1):48–60. doi: 10.1111/imr.12448 27558327

[41] Hennig-Pauka I, Imker R, Mayer L, et al. From stable to lab-investigating key factors for sudden deaths caused by *Streptococcus suis*. Pathogens. 2019;8(4):249. doi: 10.3390/pathogens8040249 31756894 PMC6963698

[42] Zhao J, Pan S, Lin L, et al. *Streptococcus suis* serotype 2 strains can induce the formation of neutrophil extracellular traps and evade trapping. FEMS Microbiol Lett. 2015;362(6):fnv022. doi: 10.1093/femsle/fnv022 25673283

[43] Auger JP, Rivest S, Benoit-Biancamano MO, et al. Inflammatory monocytes and neutrophils regulate *Streptococcus suis*-induced systemic inflammation and disease but are not critical for the development of central nervous system disease in a mouse model of infection. Infect Immun. 2020;88(3):e00787–19. doi: 10.1128/iai.00787-19 31818962 PMC7035915

[44] Chang P, Li W, Shi G, et al. The VraSR regulatory system contributes to virulence in *Streptococcus suis* via resistance to innate immune defenses. Virulence. 2018;9(1):771–782. doi: 10.1080/21505594.2018.1428519 29471718 PMC5955479

[45] de Buhr N, Stehr M, Neumann A, et al. Identification of a novel DNase of *Streptococcus suis* (EndAsuis) important for neutrophil extracellular trap degradation during exponential growth. Microbiol (Read). 2015;161(Pt 4):838–850. doi: 10.1099/mic.0.000040 25667008

[46] Liu Y, Mei J, Gonzales L, et al. Il-17a and tnf-α exert synergistic effects on expression of cxcl5 by alveolar type ii cells in vivo and in vitro. J Immunol. 2011;186(5):3197–3205. doi: 10.4049/jimmunol.1002016 21282514

[47] Theriot HM, Malaviarachchi PA, Scott MG, et al. Pulmonary expression of interleukin-17 contributes to neutrophil infiltration into the lungs during pneumonic plague. Infect Immun. 2023;91(7):e0013123. doi: 10.1128/iai.00131-23 37338372 PMC10353359

[48] Ma WT, Gu K, Yang R, et al. Interleukin-17 mediates lung injury by promoting neutrophil accumulation during the development of contagious caprine pleuropneumonia. Vet Microbiol. 2020;243:108651. doi: 10.1016/j.vetmic.2020.108651 32273025

[49] Murcia RY, Vargas A, Lavoie JP. The interleukin-17 induced activation and increased survival of equine neutrophils is insensitive to glucocorticoids. PLOS ONE. 2016;11(5):e0154755. doi: 10.1371/journal.pone.0154755 27138006 PMC4854453

[50] Zheng H. The IL-17A/neutrophils axis plays a critical role in lethal infection induced by an emerging ultra-virulent *Streptococcus suis* serotype 5 strain. Figshare. 2026. doi: 10.6084/m9.figshare.30995566.v5 PMC13290096 42308351

