# Supplementary figures and images for: The IL-17A/Neutrophil axis plays a critical role in lethal infection induced by an emerging ultra-virulent Streptococcus suis serotype 5 strain
Source: Virulence. 2026 Jun 17;17(1):2690810. doi: 10.1080/21505594.2026.2690810 (PMC13290096; doi:10.1080/21505594.2026.2690810)

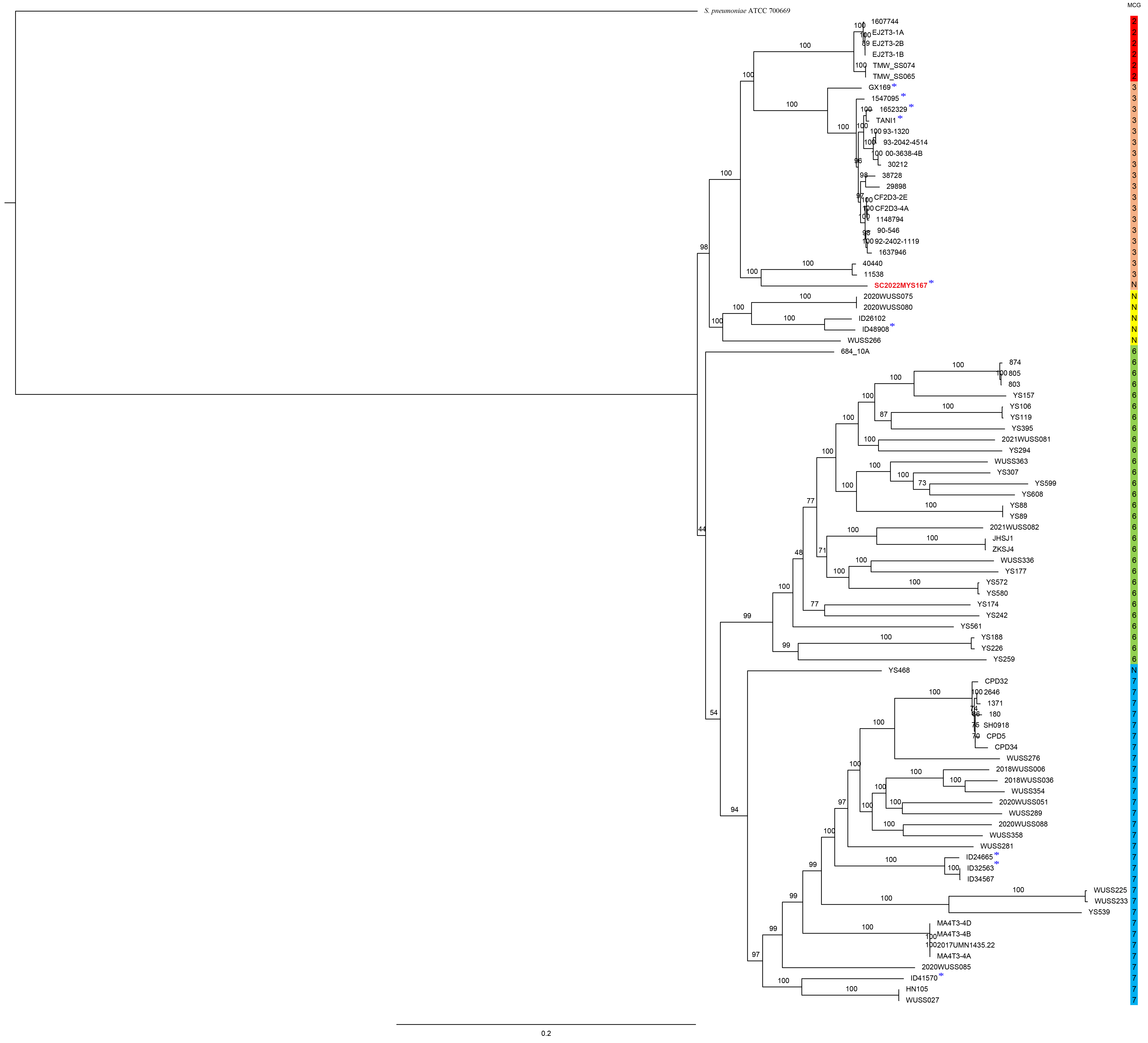

Supplement: Supplemental Material [file KVIR_A_2690810_SM6542.jpg]
