# Supplementary material for: The IL-17A/Neutrophil axis plays a critical role in lethal infection induced by an emerging ultra-virulent Streptococcus suis serotype 5 strain
Source: Virulence. 2026 Jun 17;17(1):2690810. doi: 10.1080/21505594.2026.2690810 (PMC13290096; doi:10.1080/21505594.2026.2690810)
Supplement: Supplemental Table 2.docx [file KVIR_A_2690810_SM2000.docx]

| Supplemental Table 2 Antimicrobial susceptibility of Streptococcus suis serotype 5 strain SC2022MYS167 | | | | | | | | |
| --- | --- | --- | --- | --- | --- | --- | --- | --- |
|  | Penicillin (μg/mL) | Cefazoloxime (μg/mL) | Vancomycin (μg/mL) | Clindamycin (μg/mL) | Spectinomycin (μg/mL) | Erythromycin (μg/mL) | Azithromycin (μg/mL) | Tetracycline (μg/mL) |
| Breakpoints | ≥1 | ≥8 | ＞1 | ≥1 | ≥128 | ≥1 | ≥2 | ＞8 |
| SC2022MYS167 | 0.032 | 0.5 | 0.5 | >256 | >1024 | >256 | >256 | 24 |
