# Supplementary material for: The IL-17A/Neutrophil axis plays a critical role in lethal infection induced by an emerging ultra-virulent Streptococcus suis serotype 5 strain
Source: Virulence. 2026 Jun 17;17(1):2690810. doi: 10.1080/21505594.2026.2690810 (PMC13290096; doi:10.1080/21505594.2026.2690810)
Supplement: supplementary file clean document.docx [file KVIR_A_2690810_SM1999.docx]

**Supplemental Figure 1. The maximum-likelihood phylogenetic tree of 90 *S. suis* serotype 5 genomes.** The phylogenetic tree was constructed based on the mutant SNPs in the core genomes. The *S. pneumoniae* ATCC 700669 was used as an outgroup to root the tree. * indicates genomes from patients.

**Supplemental Table 1:** **Primer sequences used in the transcriptional analysis.**

**Supplemental Table 2: The antimicrobial susceptibility profile of *S. suis* serotype 5 strain SC2022MYS167.**
