# Supplementary material for: The IL-17A/Neutrophil axis plays a critical role in lethal infection induced by an emerging ultra-virulent Streptococcus suis serotype 5 strain
Source: Virulence. 2026 Jun 17;17(1):2690810. doi: 10.1080/21505594.2026.2690810 (PMC13290096; doi:10.1080/21505594.2026.2690810)
Supplement: Supplemental Table 1.docx [file KVIR_A_2690810_SM1998.docx]

| Supplemental Table 1:Primer sequences used in the transcriptional analysis | | | |
| --- | --- | --- | --- |
| Gene | Forward Sequence | Reverse Sequence | References |
| GADPH | CCCGTAGACAAAATGGTGAAG | GACTGTGCCGTTGAATTTG | [1] |
| TLR1 | CACAGCTCCTTGGTTTTAATG | TGGGTATAGGACGTTTCTGTAG | [1] |
| TLR2 | TGGAGCATCCGAATTGCATCACCG | GAGCGGCCATCACACACCCC | [1] |
| TLR6 | CCGTCAGTGCTGGAAATAG | CGATGGGTTTTCTGTCTTGG | [1] |
| NOD1 | AGCGCCCTGTCCTTCGTCCT | GCACCTTCACCCCCGTGTCG | [2] |
| NLRP1 | GCTGAATGACCTGGGTGATGGT | CTTGGTCACTGAGAGATGCCTG | [3] |
| NLRP3 | ATTACCCGCCCGAGAAAGG | TCGCAGCAAAGATCCACACAG | [4] |
| NLRC4 | TGGCCCTAGAAGGTGTGTGTTC | GAGGAGCCCTATTGTCACCA | [3] |
| AIM2 | GTACCGGGAAATGCTGTTGT | TCCTGGCAATCTGAAACTCA | [3] |
| CD11b | GCCAGAACCCGCTCACCAA | TTCAGAGCCCCATGCCCTT | [5] |
| F4/80 | CTTTGGCTATGGGCTTCCAGTC | GCAAGGAGGACAGAGTTTATCGTG | [6] |
| Ly6g | GACTTCCTGCAACACAACTACC | ACAGCATTACCAGTGATCTCAGT | [7] |
| **References** | | | |
| [1] Zheng H, Punaro MC, Segura M, et al. Toll-like receptor 2 is partially involved in the activation of murine astrocytes by Streptococcus suis, an important zoonotic agent of meningitis. J Neuroimmunol. 2011;234(1-2):71-83. doi:10.1016/j.jneuroim.2011.02.005 | | | |
| [2] Qi KX, Yi XL, Wang ML, et al.Characteristics and mechanism of cerebral inflammatory response induced by Streptococcus parasuis clinical strains in mice.Disease surveillance,2023,38(03):351-357.DOI:10.3784/jbjc.202301020558. | | | |
| [3] Quan JH, Gao FF, Ma TZ, et al. Toxoplasma gondii Induces Pyroptosis in Human Placental Trophoblast and Amniotic Cells by Inducing ROS Production and Activation of Cathepsin B and NLRP1/NLRP3/NLRC4/AIM2 Inflammasome. Am J Pathol. 2023;193(12):2047-2065. doi:10.1016/j.ajpath.2023.08.016 | | | |
| [4] Sun L, Yong Y, Wei P, et al. Electroacupuncture ameliorates postoperative cognitive dysfunction and associated neuroinflammation via NLRP3 signal inhibition in aged mice. CNS Neurosci Ther. 2022;28(3):390-400. doi:10.1111/cns.13784 | | | |
| [5] Yamanishi K, Mukai K, Hashimoto T, et al. Physiological and molecular effects of interleukin-18 administration on the mouse kidney. J Transl Med. 2018;16(1):51. Published 2018 Mar 7. doi:10.1186/s12967-018-1426-6 | | | |
| [6] Nasiri-Ansari N, Nikolopoulou C, Papoutsi K, et al. Empagliflozin Attenuates Non-Alcoholic Fatty Liver Disease (NAFLD) in High Fat Diet Fed ApoE(-/-) Mice by Activating Autophagy and Reducing ER Stress and Apoptosis. Int J Mol Sci. 2021;22(2):818. Published 2021 Jan 15. doi:10.3390/ijms22020818 | | | |
| [7] Yu M, Pan H, Che N, et al. Microwave ablation of primary breast cancer inhibits metastatic progression in model mice via activation of natural killer cells. Cell Mol Immunol. 2021;18(9):2153-2164. doi:10.1038/s41423-020-0449-0 | | | |
